# The Aryl Hydrocarbon Receptor: Differential Contribution to T Helper 17 and T Cytotoxic 17 Cell Development

**DOI:** 10.1371/journal.pone.0106955

**Published:** 2014-09-09

**Authors:** Mark D. Hayes, Vitalijs Ovcinnikovs, Andrew G. Smith, Ian Kimber, Rebecca J. Dearman

**Affiliations:** 1 Faculty of Life Sciences, The University of Manchester, Manchester, United Kingdom; 2 MRC Toxicology Unit, The University of Leicester, Leicester, United Kingdom; University of São Paulo, Brazil

## Abstract

The aryl hydrocarbon receptor (AhR) has been shown to be required for optimal Thelper (Th) 17 cell activation. Th17 cells provide immunity against extracellular pathogens and are implicated in autoimmune diseases. Herein, the role of the AhR in cytokine production by Th17, and by the analogous population of T cytotoxic (Tc)17 cells, has been examined. Lymph node Tc (CD8^+^) and Th (CD4^+^) cells were isolated by negative selection from naive AhR^+/−^ and AhR^−/−^ mice and polarised to Tc1/Th1 or Tc17/Th17 phenotypes with appropriate cytokines. Cell differentiation was assessed as a function of mRNA and protein (ELISA and flow cytometry) expression for interferon (IFN)-**γ** and for key Th17 cytokines. In AhR^+/−^ mice, Th17 cells displayed an exclusive IL-17 profile, which was markedly inhibited by a selective AhR antagonist to levels observed in AhR knockout mice. Addition of the natural AhR agonist 6-formylindolo[3,2-b]carbazole (FICZ) markedly enhanced Th17 cell activity in the heterozygotes. In contrast, Tc17 cells polarised into 3 distinct subsets: producing either IL-17 or IFN-γ alone, or both cytokines. Blocking AhR was also detrimental to Tc17 development, with reduced responses recorded in AhR^−/−^ mice and antagonist-mediated reduction of IL-17 expression in the heterozygotes. However, Tc17 cells were largely refractory to exogenous FICZ, presumably because Tc17 cells express baseline AhR mRNA, but unlike Th17 cells, there is no marked up-regulation during polarisation. Thus, Th17 cell development is more dependent upon AhR activation than is Tc17 cell development, suggesting that endogenous AhR ligands play a much greater role in driving Th17 cell responses.

## Introduction

An important property of adaptive immune function is the orchestration of polarised immune responses by the generation of discrete populations of CD4^+^ T helper (Th) and CD8^+^ cytotoxic T (Tc) lymphocytes [1], [2]. A more recent addition to known subsets of CD4^+^ Th cells are Th17 cells that have a characteristic cytokine secretion profile that includes IL-17A, IL-17F, IL-21 and IL-22. These cells play important roles in protective immunity and have been implicated in the pathogenesis of certain autoimmune diseases including multiple sclerosis, psoriasis, rheumatoid arthritis and Crohn's disease [3]–[5]. The effector functions of Th17 cells are cytokine-mediated. Proinflammatory IL-17A acts as a potent neutrophil recruiter, as well as stimulating other neutrophil-attracting and activating cytokines and chemokines, and is an important component of resistance to pathogenic microorganisms [6]. IL-22 targets non-hematopoietic cells (keratinocytes, hepatocytes and colonic epithelial cells) inducing proliferative and anti-apoptotic pathways and the production of anti-microbial molecules that aid tissue repair [7]. In autoimmunity, Th17 cytokines are thought to enhance inflammation by paracrine induction of other proinflammatory factors, such as tumour necrosis factor-α and IL-6 [8], [9].

In addition to the various CD4^+^ Th cell subsets, CD8^+^ Tc cells analogous with Th1 and Th2 cells have been described both *in vitro* and *in vivo*, and have been categorised as Tc1 and Tc2 cells, respectively [10]. The most recent addition to the family are CD8^+^ cells secreting IL-17, and classified as Tc17 [11]. It has been claimed previously that Tc17 cells are an *in vitro* artefact due to their lack of cytotoxic activity, associated with the absence of perforin and Granzyme B[12]. However, more recent studies have provided evidence for the existence of Tc17 cells in both mouse and humans [13]–[15]. Although Tc17 cells express cytokine profiles similar to their CD4^+^ counterparts, their roles in protective immunity and autoimmune disease have yet to be established. An interesting characteristic of both Th17 and Tc17 cells is their plasticity. The switch from Th17 to Th1 phenotype has been shown *in vivo* using Th17 reporter mice and a range of inflammatory and autoimmune conditions. For example, the majority of Th1 cells that had infiltrated spinal tissue during the development of experimental autoimmune encephalomyelitis had at some time previously expressed IL-17A, thus demonstrating that they had derived from Th17 cells [16]. Tc17 cells have also been shown to display plasticity. Tc17 cells generated *ex vivo* were found to switch off IL-17 production when transferred into mice, and interestingly, this coincided with the acquisition of cytotoxic ability, even in the absence of interferon (IFN)-γ production [17].

The conditions for Th17 development have been investigated thoroughly and although there are similar requirements for Tc17 development, there may also be some differences. Th17 and Tc17 polarisation have both been shown to require transforming growth factor (TGF)-β and IL-6, and to be enhanced further by IL-1β, IL-21 and IL-23 [18], [19]. In addition, it has been shown that activation of the aryl hydrocarbon receptor (AhR) is required for optimal Th17 polarisation. The AhR was first described as a receptor for ligands that are environmental toxicants, such as 2,3,7,8-tetrachlorodibenzo-p-dioxin (TCDD) or dioxin [20]. This receptor is a cytoplasmic transcription factor that following ligation translocates to the nucleus where it binds to the AhR nuclear translocator forming a heterodimer that can activate various AhR responsive genes [20], [21]. AhR ligands fall into two categories: synthetic and natural. Although initial characterisation of AhR focused primarily on TCDD and other synthetic halogenated hydrocarbons, more recently ligation by natural ligands and the role of AhR in immune function has attracted increasing interest. Natural ligands include plant-derived materials, such as flavonoids and by-products of dietary indoles, such as 6-formylindolo[3,2-b]carbazole (FICZ) that is a photoproduct of tryptophan [22]. Although the expression of AhR is ubiquitous in vertebrate cells [23], so far there are only two known conventional T cell populations that actively up-regulate this receptor when activated: regulatory T cells and Th17 cells [24]. However, to date there have been no investigations reported of the involvement of the AhR in Tc17 development.

The similarity of Tc17 cells to their CD4^+^ counterparts with respect to their requirements for polarisation and cytokine expression profiles has led us to hypothesise that activation of the AhR may impact on Tc17 phenotype. We have therefore investigated the contribution of the AhR to Tc17 polarisation *in vitro*. In addition, comparisons have been made between Th17 and Tc17 development in T cells obtained from AhR knockout mice. Successful polarisation has been measured as a function of frequency of IL-17A-expressing cells by flow cytometry, and at the levels of mRNA and secreted protein for IL-17A and IL-22.

## Materials and Methods

### Animals

AhR^−/−^ and AhR^+/−^ mice on a C57BL/6J background, originally obtained from the Jackson Laboratory via B. Stockinger (National Institute for Medical Research, London, UK), were bred in the specific pathogen free facility at the University of Manchester. The AhR transgenic mice were originally derived from 129X1 x 129S1 via R1 (+Kitl-SlJ) ES cell line and donated to the Jackson Laboratory by Dr. Christopher Bradfield (University of Wisconsin Medical School). Mice were bred from male homozygous (AhR^−/−^) and female heterozygous (AhR^+/−^) breeding pairs.

### Ethics statement

All animal procedures were approved by the UK Home Office and carried out in compliance with the Animals (Scientific Procedures) Act 1986 under a Home Office granted project licence. Mice were provided with environmental stimuli (bedding and nesting materials); with food (Beekay Rat and Mouse Diet No1 pellets; B&K Universal, Hull, UK) and water being available *ad libitum.* The ambient temperature was maintained at 21±2°C and relative humidity was 55±10% with a 12 h light/dark cycle. Mice were sacrificed by exposure to CO_2_ gas in rising concentration followed by dislocation of the neck in concordance with schedule 1 (Animals [Scientific Procedures] Act 1986).

### Genotyping of AhR mice

Genotyping was performed with two sets of PCR primers (Sigma Aldrich, Gillingham, UK). The first amplified a region of AhR exon 2 present in the wild type but not the knockout mice, that is, forward TTCAGAGTAAAGCCCATCCC and reverse ATCAAAGAAGCTCTTGGCCC. The second set amplified a region of the neomycin gene only present in the knockout animals, that is, forward TGGGTGGAGAGGCTATTC and reverse ATGGTGAGATGACAGGAGATC (http://blast.ncbi.nlm.nih.gov/). Mice were genotyped prior to experimentation using DNA derived from ear clip tissue.

### Th1/Tc1 and Th1/Tc17 polarisation

CD4^+^ and CD8^+^ T cells were isolated from skin draining lymph nodes (LN: axillary, inguinal and auricular) by magnetic separation (Stemcell Technologies, Manchester, UK). The culture medium for both Th1/Tc1 and Th17/Tc17 polarisation was IMDM (Sigma), supplemented with 25 mM HEPES, 400 µg/ml streptomycin/penicillin, 292 µg/ml L-Glutamine, 5 mM 2-mercaptoethanol and 5% heat-inactivated fetal calf serum (v/v) (all from Life Technologies, Paisley, UK). A total of 2.5×10^5^ T cells/well were differentiated in wells coated with 1 µg/ml anti-CD3 plus 10 µg/ml anti-CD28 (eBioscience, Hatfield, UK) with a cytokine cocktail of 50 ng/ml human IL-6, 1 ng/ml human TGF-β, and 10 ng/ml IL-1β for Th17/Tc17 polarisation and 3 ng/ml IL-12 for Th1/Tc1 polarisation (all cytokines from R & D systems, Abingdon, UK). The AhR antagonist CH-223191 (Calbiochem, Nottingham, UK) or AhR agonist FICZ (Enzo Life sciences, Exeter, UK) were added at 3 µM and 300 nM respectively. Both were formulated in dimethyl sulfoxide (DMSO) at the start of culture with control wells received appropriate amounts of DMSO.

### RT-PCR

Total RNA was purified from enriched CD4^+^ or CD8^+^ cells using TRIzol and Purelink RNA mini kit (Life Technologies). The mRNA expression levels of mouse IL-17A, IL-17F, IFN-γ, T-bet, RORC, AhR, AhRR, Cyp1A1 genes were determined by RT-PCR using Taqman primers (Life Technologies) on a RT-PCR machine (StepOne plus; Life Technologies). Expression was normalised using freshly isolated unpolarised cells (control) and to hypoxanthine–guanine phosphoribosyl transferase (HPRT), with the ΔΔ cyclic threshold (Ct) method used to calculate relative fold change.

### Flow cytometry

Single cell suspensions of freshly isolated CD8^+^ enriched cells were characterised for memory/effector phenotype by flow cytometry. Cells were treated with 5 µg/ml anti-CD16/32 blocking antibody prior to staining with specific antibodies; 5 µg/ml fluorescein isothiocyanate (FITC) labelled anti-CD8 (eBioscience), 2 µg/ml allophycocyanin (APC) labelled anti-127 (eBioscience), 2 µg/ml phycoerythin (PE) labelled anti-CD62L (Becton Dickinson, Oxford, UK), or with appropriate isotype controls. Cells were defined as follows: naïve, single positive CD62L; central memory, double positive; effector memory, single positive CD127; effector, double negative [25]. In addition, the frequency of cytokine expressing cells was characterised by flow cytometry. Single cell suspensions of *in vitro* polarised T cells were treated with phorbol 12,13-dibutyrate (PdBu; 500 ng/ml), ionomycin (500 ng/ml) and 10 µg/ml brefeldin A for 4 h (Sigma). Following restimulation cells were fixed and permeabilised using 2% paraformaldehyde and saponin (0.1% w/v) (Sigma). Blocking antibodies were applied prior to staining for specific antibodies; 10 µg/ml anti-CD16/32, 10 µg/ml hamster IgG and 10 µg/ml rat IgG (BD, Oxford, UK). Cells were then stained with 2.5 µg/ml PE labelled anti-IL-17A and 10 µg/ml APC labelled anti-IFN-γ and appropriate isotype controls (Biolegend, San Diego, CA, USA). In each case, acquisition of samples (25000 cells) was performed on a flow cytometer (FACScalibur; Becton Dickinson) and data were analysed using FlowJo v7.5 software (Tree Star, Ashland, OR, USA). For memory effector phenotype, cells were initially gated on forward and side scatter and non-viable cells excluded using 7-aminoactinomycin D (7-AAD; 1 µg/ml). Cells that stained positive for CD8, which constituted 84–96% of the total enriched population, were analysed for memory/effector marker expression. Cytokine expressing cells were initially gated on forward scatter and side scatter. In both cases, gates were drawn based upon the position of cells stained with isotype controls, with >99% of the double isotype control stained cells residing in the lower left hand quadrant (Th1/Tc1 gating strategy supplied as Figure S1).

### Cytokine determinations using ELISA

The cytokines IL-17A, IL-22 and IFN-γ were analysed in supernatants using DuoSet ELISA kits (R&D systems) following the manufacturers' instructions. Optical density at 450 nm was measured using an automated reader (Multiskan, Thermo scientific, Basingstoke, UK). The lower limit of accurate detection was approximately 25–50 pg/ml. Standard errors were less than 10% in most experiments.

### Statistical analyses

Statistical significance of differences between groups was determined using one-way ANOVA with a Tukey post test using Prism 5 software (GraphPad Software, La Jolla, CA, USA). Significant differences are illustrated by * p<0.05, ** p<0.01 and *** p<0.001.

## Results

### *In vitro* polarised Th17 and Tc17 cells display different cytokine profiles

**Figure 1:**
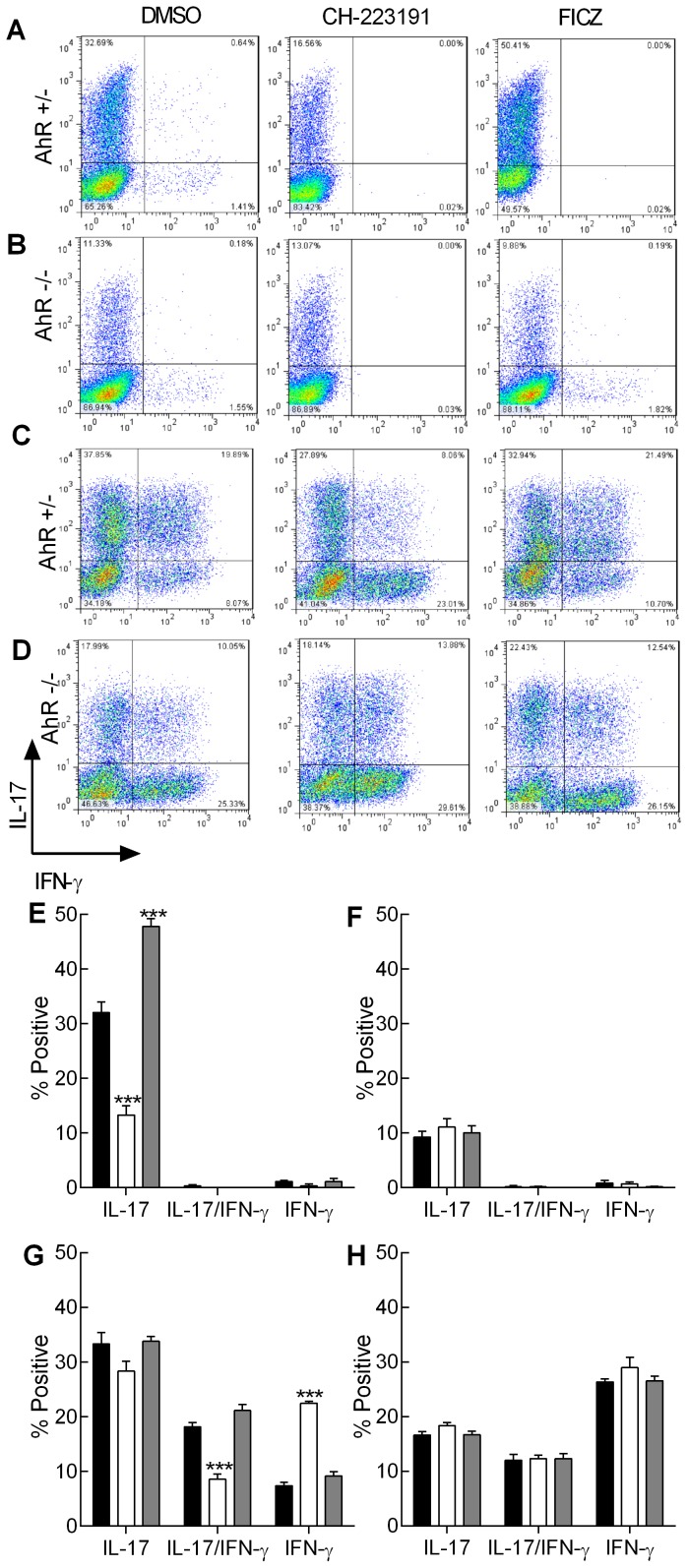
The effect of AhR modulation on Th17 and Tc17 cell frequency. Naïve CD4^+^ cells from AhR^+/−^ (A and E) or AhR^−/−^ mice (B and F) and naïve CD8^+^ cells from AhR^+/−^ (C and G) or AhR^−/−^ mice (D and H) were polarised under Th17/Tc17 conditions for 5 days. Cells were then stimulated with PdBU and ionomycin in the presence of brefeldin A. The polarised cells were then permeabilised and stained using fluorescent anti-IL-17A (PE [Y-axis]) and -IFN-γ (APC [X-axis]) labelled antibodies. Cells (25000) were analysed by flow cytometry. The cells were cultured in the presence of AhR antagonist (CH-223191) (white bar) or AhR agonist (FICZ) (grey bar) both formulated in DMSO or with an equivalent amount of DMSO alone (black bar). Representative quadrant analyses are illustrated (A–D). The percentage of cells positive for IL-17, IL-17 and IFN-γ or IFN-γ alone was calculated by subtracting the isotype controls from the positive cells in each quadrant and are illustrated as % positive cells (E-H; mean ± SE; n = 3 independent experiments). Statistical significance of differences between DMSO control and AhR antagonist/agonist treated cells was analysed by one-way ANOVA. ***, *p* < 0.001.

Naïve CD4^+^ or CD8^+^ cells from both AhR^+/−^ and AhR^−/−^ mice were polarised under conditions shown previously to generate Th17 cells (medium known to contain AhR agonists; IMDM) [24], [26]. Following 5 days in culture the extent of successful polarisation was analysed as a function of intracellular cytokine expression by flow cytometry, cytokine mRNA levels by RT-PCR and secreted cytokine by ELISA. The contribution of the AhR was assessed by either blocking the AhR with a selective antagonist (CH-223191) or by addition of excess natural agonist (FICZ). In the control vehicle treated group, Th17 cells generated from CD4^+^ precursors from AhR^+/−^ mice were similar to those published previously, with approximately 30% of cells expressing intracellular IL-17 (Figure 1A, E). This frequency was significantly reduced (by ∼50%) in the presence of the AhR antagonist. Conversely this population was markedly enhanced by the addition of exogenous FICZ (Figure 1A, E). When Th17 cells were generated from CD4^+^ precursors from AhR^−/−^ mice, the maximum yield of IL-17^+^ cells varied between ∼10–14%, and was unaffected by the addition of the agonist or antagonist (Figure 1B, F). Analysis of IFN-γ production revealed that independent of treatment or genotype, <2% of cells produced the Th1 cytokine. Further, no cells expressed both cytokines. However, CD8^+^ cells polarised under identical conditions displayed significantly different phenotypes. CD8^+^ cells cultured under Th17 conditions displayed 3 distinct cytokine profiles; those that expressed only IL-17 or IFN-γ or those that expressed both cytokines. In cells from AhR^+/−^ mice, the frequency of the Tc17 population was similar to the relative proportion of Th17 cells (Figure 1C, G). However unlike Th17 cells, there was no impact of AhR agonist or antagonist (∼30% of cells were IL17^+^ irrespective of treatment). The population that was most susceptible to the addition of the AhR ligands was the IL-17/IFN-γ fraction, where a ∼50% reduction was observed following addition of the antagonist but the agonist was without effect (Figure 1C, G). Interestingly, the reduction in the double positive population coincided with a reciprocal increase in the IFN-γ single positive population. CD8^+^ cells from AhR^−/−^ mice polarised into 3 distinct populations similar to those found in the heterozygotes. However, the frequency of the single positive IL-17 cells remained around 20% under all conditions, and the double positive IL-17/IFN-γ and single positive IFN-γ populations remained approximately 10% and 25% respectively (Figure 1D, H). Thus, for the CD8^+^ dual expressing cells and the single positive IFN-γ populations, cell frequencies recorded for AhR^+/−^ derived cells in the presence of antagonist were equivalent to those recorded for the AhR knockout mice. In contrast, frequencies of single IL-17 expressing AhR^+/−^ cells were somewhat higher than in the AhR^−/−^ mice, suggesting that this population is less sensitive to the antagonist, at least using this end point.

It has been suggested that the influence of AhR on CD8 cells is dependent upon the maturation status of the cell [27]. Therefore in order to rule out potential skewing of responses due to differential numbers of effector versus naïve cells in the starting CD8 cell population, the frequency of naive, central memory, effector memory and effector cells was characterised by flow cytometry (Table 1). In both strains of mice, fewer than 2% of cells were of the effector memory or effector cell phenotype; the majority of cells were central memory cells (∼60%) and the remainder (∼40%) displayed a naïve phenotype, regardless of the AhR genotype. These starting cell populations were also assessed for cytokine expression by flow cytometry: consistent with the lack of CD8 effector cells, there was no detectable IL-17 or IFN-γ expression by these initial populations of cells (data not shown). The cells were also cultured under Tc0 conditions (anti-CD3 and anti-CD28 but in the absence of polarising cytokines) for 5 days and the frequency of cytokine expressing cells enumerated by flow cytometry. Under these conditions, ∼15% IFN-γ only expressing cells and <1% double positive cells were recorded for cells isolated from either AhR^+/−^ or AhR^−/−^ mice (Table 1), and there was no impact of either antagonist or FICZ on these frequencies (data not shown). Taken together, these data demonstrate that not only is there is no detectable difference between the two mouse strains with respect to the distribution of naive/memory/effector cells in the starting population but also that it is unlikely that precommitted CD8 effector cells are contributing to the observed phenotype.

**Table 1:** Influence of AhR genotype on the memory/effector phenotype of the precursor CD8+ cells (a) and upon the Th0 cytokine profile (b).

(a)	Cell frequency (mean and SE)			
	Naïve	Central memory	Effector memory	Effector
AhR^+/−^	39.5+/−1.5	56.4+/−2.6	2.0+/−0.7	2.1+/−0.7
AhR^−/−^	41+/−4.1	55.0+/−4.2	1.6+/−0.2	2.2+/−0.3

(b)	Percentage cytokine expressing cells (mean and SE)		
	IFN-γ	IL-17	IFN-γ/IL-17
AhR^+/−^	10.6+/−2.7	0.1+/−0.1	0
AhR^−/−^	16.6+/−4.2	0.6+/−0.2	0

Naïve CD8^+^ cells from AhR^+/−^ or AhR^−/−^ mice (n = 3) were isolated and the frequency of naïve, central memory, effector memory and effector cells was characterised by flow cytometry (a). Cells were cultured under Th0 conditions for 5 days. Cells were then stimulated with PdBU and ionomycin in the presence of brefeldin A. The polarised cells were then permeabilised and stained using fluorescent anti-IL-17A (PE [Y-axis]) and -IFN-γ (APC [X-axis]) labelled antibodies and appropriate isotype control labelled antibodies (b). In each case, cells (25000) were analysed by flow cytometry.

**Figure 2:**
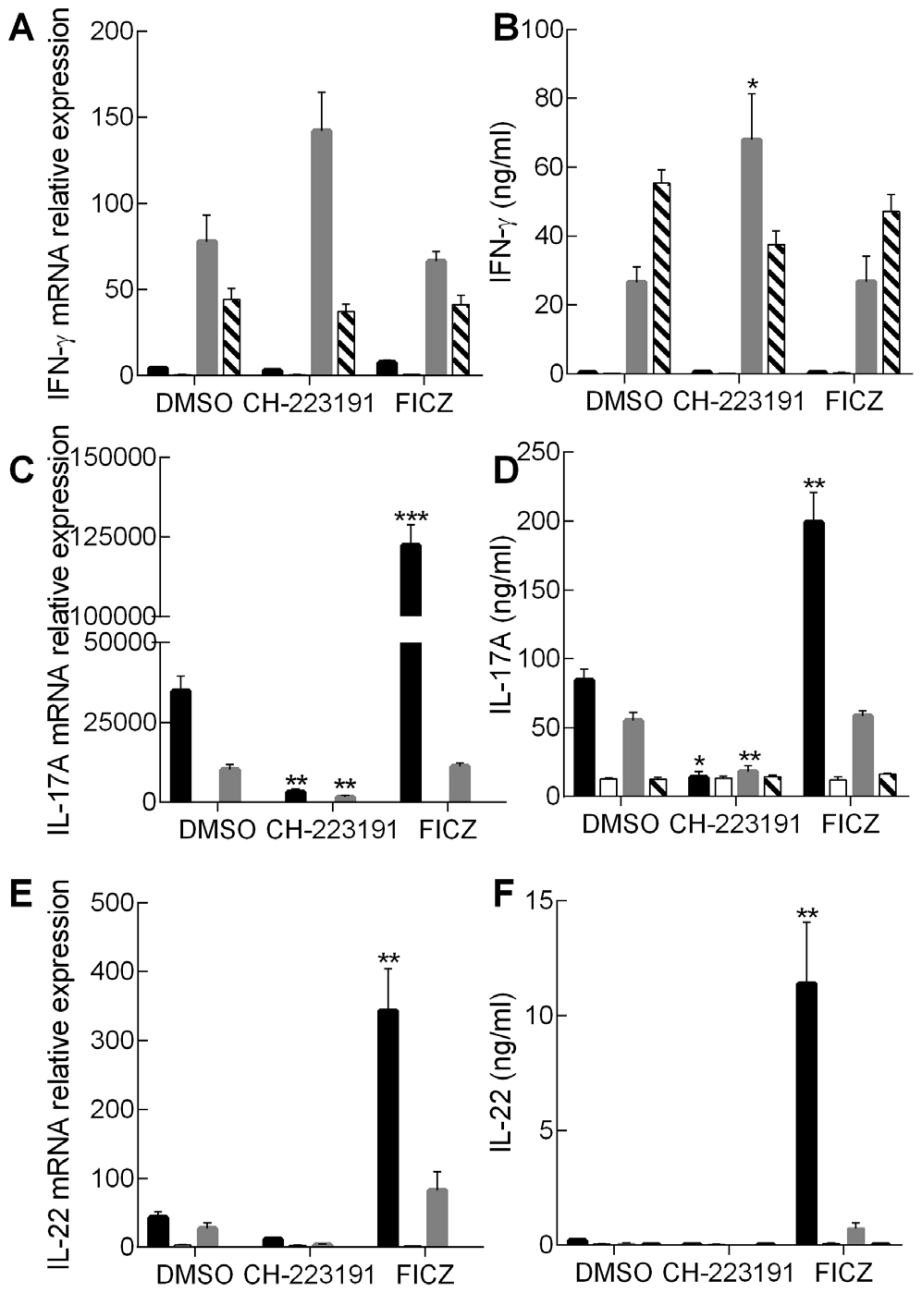
Cytokine mRNA and protein expression profiles of Th17 and Tc17 cells: effect of AhR modulation. Naïve CD4^+^ cells from AhR^+/−^ (black bar) or AhR^−/−^ mice (white bar) and naïve CD8^+^ cells from AhR^+/−^ (grey bar) or AhR^−/−^ mice (striped bar) were polarised under Th17/Tc17 conditions for 5 days in the presence of AhR antagonist (CH-223191) or AhR agonist (FICZ) both formulated in DMSO or with an equivalent amount of DMSO alone. Cells were harvested and total RNA prepared for the analysis of mRNA levels for IL-17A, IL-22, and IFN-γ using RT-PCR and the ΔΔ Ct method (A, C and E). Results were normalised against naive CD4^+^ or CD8^+^ cells and the housekeeping gene HPRT. Supernatants were also analysed for secreted cytokine by ELISA (B, D and F). Results are shown as mean ± SE for n = 3 independent experiments. Statistical significance of differences between DMSO control and AhR antagonist/agonist treated cells was analysed by one-way ANOVA. *, *p* < 0.05; **, *p* < 0.01; ***, *p* < 0.001.

In parallel with measuring the frequency of cytokine expressing cells, *in vitro* polarised cells were analysed for IL-17, IL-22 and IFN-γ expression at the level of mRNA by RT-PCR and cytokine secretion by ELISA. The pattern of IL-17 mRNA and protein secretion paralleled closely the results seen for intracellular cytokine staining, with expression by AhR^+/−^ Th17 cells significantly reduced by the antagonist and enhanced by the addition of FICZ (Figure 2A, B). Furthermore, the AhR^+/−^ Th17 cells were the only population that expressed significant amounts of IL-22 and then only in response to exogenous FICZ. In contrast, production of both IL-17 and IL-22 by AhR^−/−^ Th17 cells was reduced to levels to those recorded for AhR^+/−^ Th17 cells following treatment with the antagonist (Figure 2); with these relatively low levels unaffected by addition of either ligand. In addition, neither heterozygous nor knockout Th17 cells expressed significant IFN-γ (Figure 2E, F). The levels of IL-17 expressed by Tc17 cells from AhR^+/−^ mice were markedly lower than those produced by Th17 cells from the same mice. Tc17 IL-17 production at the level of both message and secreted cytokine was inhibited by the presence of the antagonist whereas addition of exogenous FICZ was without effect (Figure 2A, B). As noted for the AhR^−/−^ Th17 cells, Tc17 cells derived from the same mice expressed relatively low levels of IL-17, again comparable with the amounts produced when the AhR^+/−^ Tc17 cells were cultured with the antagonist. This suggests that the lack of effect of the antagonist on the frequency of single positive IL-17 CD8 cells detected by flow cytometry is not functionally relevant. For the majority of Tc17 cultures, IL-22 production was low or undetectable, with the exception of AhR^+/−^ Tc17 cells cultured in the presence of exogenous FICZ. However, this was still dramatically lower than the amount expressed by Th17 cells from the same mice cultured under the same conditions. Although the Th17 cell populations did not express IFN-γ, all CD8^+^ cells cultured under Tc17 conditions produced this cytokine. Treating the AhR^+/−^ CD8^+^ cells with the antagonist enhanced IFN-γ expression, whereas FICZ was without effect (Figure 2E, F). Vigorous IFN-γ expression was recorded for AhR^−/−^ CD8^+^ cells which was largely unaffected by AhR ligands.

### Kinetics of polarisation reveals differences between Th17 and Tc17 development

**Figure 3:**
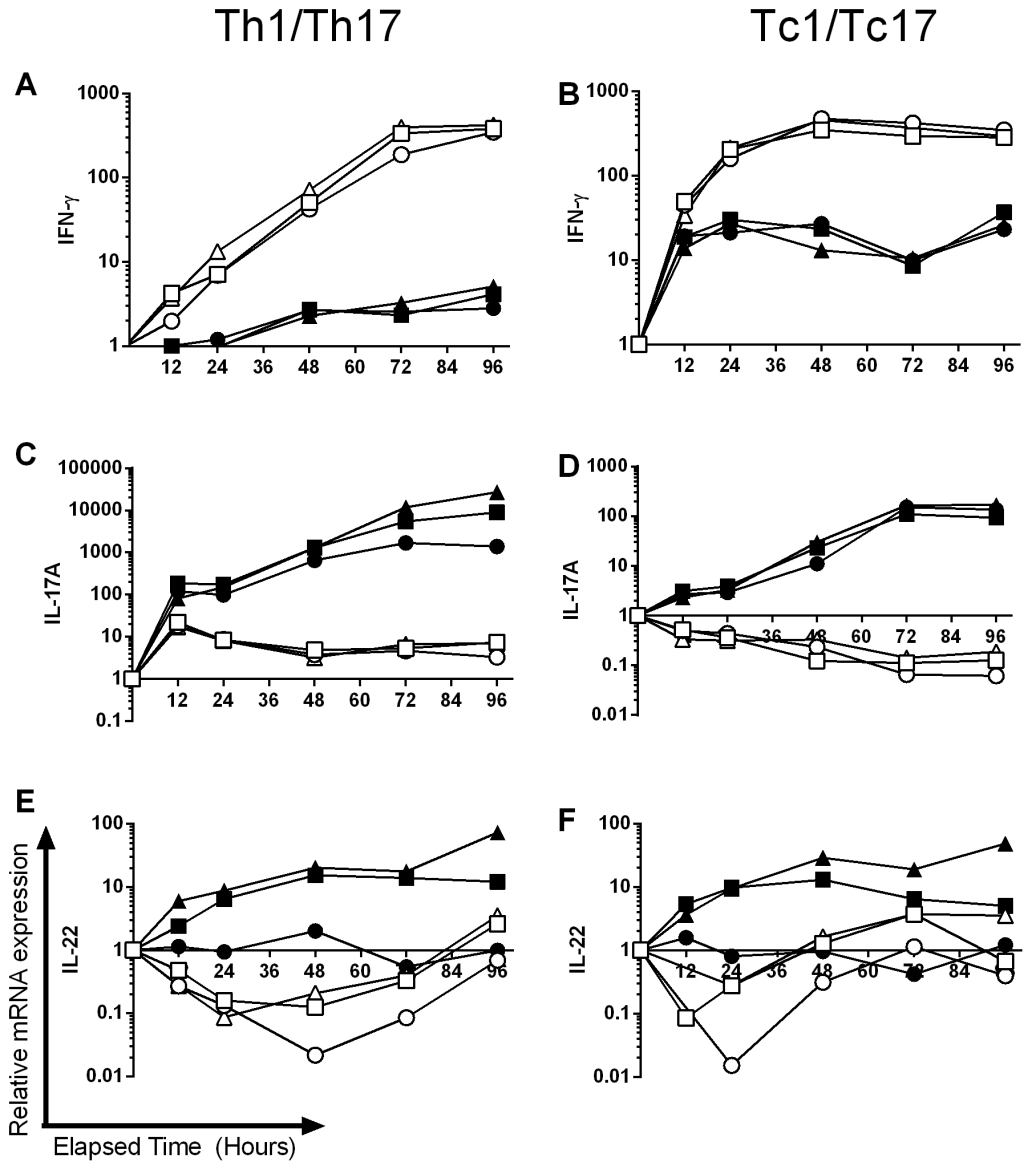
The kinetics of cytokine expression during Th1/Tc1 and Th17/Tc17 polarisation. Naïve CD4^+^ or CD8^+^ cells from AhR^+/−^ mice were cultured for 96 h under Tc1/Th1 (open symbols) or Th17/Tc17 (closed symbols) polarising conditions in the presence of 3 µM CH-223191 (•,○) or 300 nM FICZ (▴,Δ) formulated in DMSO or with an equivalent amount of DMSO alone (▪,□). At selected time points (12–96 h) RNA was isolated and levels of IFN-γ (A, B), IL-17 (C, D) and IL-22 (E, F) mRNA transcripts were analysed by RT-PCR using the ΔΔ Ct method. Results were normalised against naive CD4^+^ or CD8^+^ cells and the housekeeping gene HPRT. A single experiment is shown as a function of relative mRNA expression on a logarithmic scale.

To characterise potential differences between Th17 and Tc17 development, the kinetics of expression of a panel of genes was examined. Th1 and Tc1 cells were also analysed, the former being known to develop independently of AhR [24]. The expected Th1 and Tc1 patterns of cytokine expression and lack of impact of AhR ligands were confirmed in day 5 polarised cells isolated from AhR heterozygous and knockout mice (Figures S2–S4). Subsequently, the kinetics of IL-17, IL-22 and IFN-γ expression in Th1/Th17 and Tc1/Tc17 cells derived from AhR heterozygous mice was examined. As expected, expression of IFN-γ was highest in Th1 and Tc1 populations, with no impact of the AhR antagonist or FICZ treatment. IFN-γ levels were lower in the Tc17 populations and reduced further still in the Th17 group (Figure 3A, B). Conversely, analysis of IL-17 expression levels revealed that the highest levels were achieved for Th17 cells, then Tc17 cells, with FICZ treatment increasing IL-17 expression only for Th17 cells, and the antagonist decreasing cytokine expression in each case (Figure 3C, D). The pattern of IL-22 expression was very similar for Th17 and Tc17 cells, with very profound inhibition by the antagonist recorded (Figure 3E, F). Interestingly, whereas Tc17 cells expressed relatively high levels of IFN-γ, levels of IL-17A, and to some extent IL-22, were markedly down-regulated in Tc1 cells, to levels considerably lower than those recorded in the naïve precursor CD8^+^ cell population.

**Figure 4:**
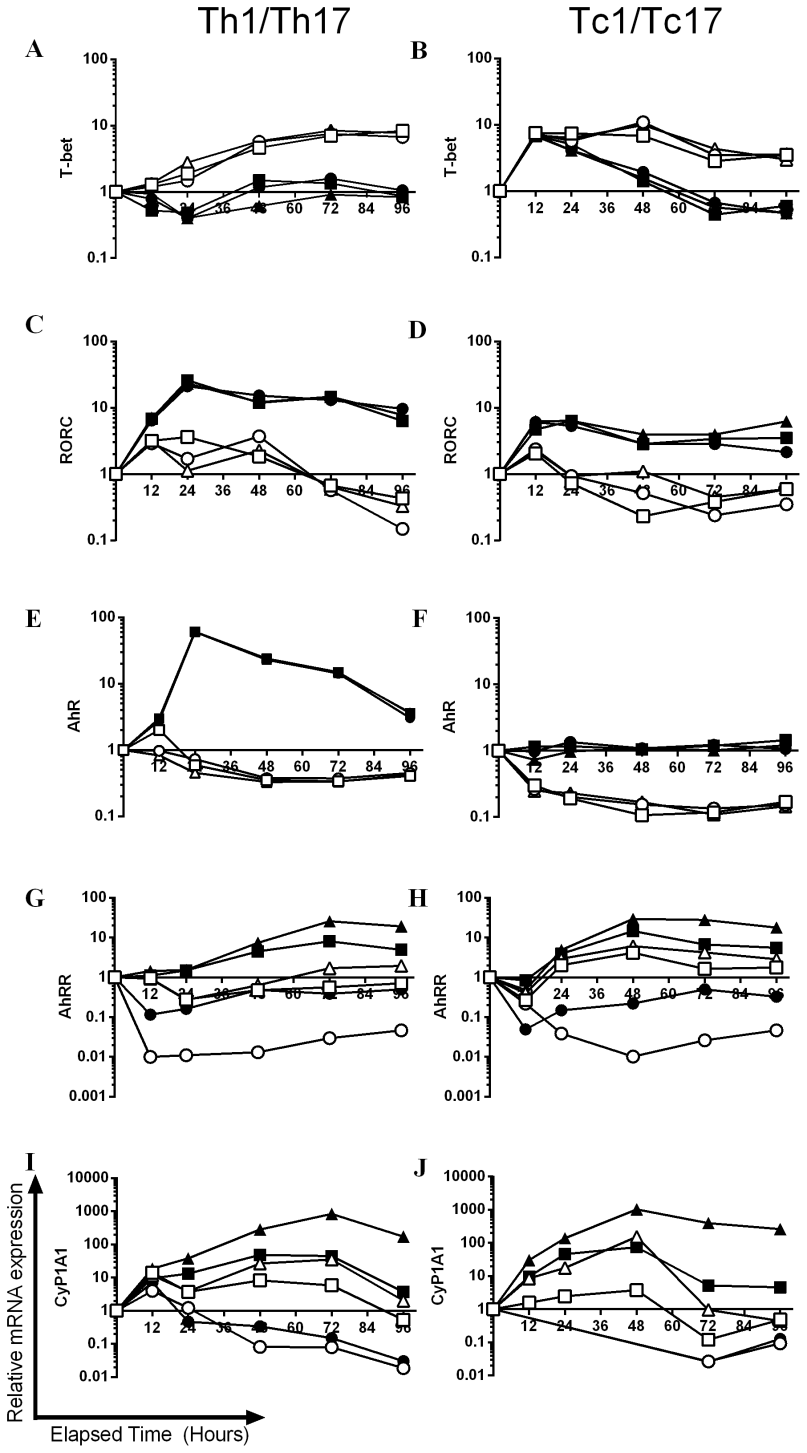
The kinetics of expression of master transcription factors and AhR associated genes during Th1/Tc1 and Th17/Tc17 polarisation. Naïve CD4^+^ or CD8^+^ cells from AhR^+/−^ mice were cultured for 96 h under Tc1/Th1 (open symbols) or Th17/Tc17 (closed symbols) polarising conditions in the presence of presence of 3 µM CH-223191 (•,○) or 300 nM FICZ (▴,Δ) formulated in DMSO or with an equivalent amount of DMSO alone (▪,□). At selected time points (12–96 h) total RNA was isolated and levels of mRNA transcripts for RORC (A, B), T-bet (C, D), AhR (E, F), AhRR (G, H) and Cyp1A1 (I, J) were analysed by RT-PCR using the ΔΔ Ct method. Results were normalised against naive CD4^+^ or CD8^+^ cells and the housekeeping gene HPRT. A single experiment is shown as a function of relative mRNA expression on a logarithmic scale. If expression levels were too low to achieve a meaningful CT value for a given sample, due to the logarithmic scale, these data were not illustrated graphically (12–48 h CH223191 data in J).

Finally, genes associated with AhR activation (AhRR; the AhR repressor and Cyp1A1) and expression levels of the AhR itself were analysed. Similar to previous reports [26], the AhR was up-regulated under Th17 polarising conditions, whereas Th1 or Tc1 stimulation resulted in active down-regulation (Figure 4E, F). Importantly, there was no up-regulation of AhR under Tc17 polarising conditions, with expression maintained at baseline levels (Figure 4F). Expression of AhRR, which is stimulated in response to AhR activators forming a negative feedback loop [28], was remarkably similar between CD4^+^ and CD8^+^ cell polarisations. The highest levels of this gene were recorded under Th17/Tc17 conditions in the presence of FICZ. While AhRR expression in all cells, regardless of polarising conditions, was down-regulated by the antagonist; the most marked effects were recorded for Th17/Tc17 cells (Figure 4G, H). As Cyp1A1 has long been used as a biomarker of AhR activation and also forms part of the negative feedback loop by metabolising AhR ligands [29], the kinetics of its expression was also investigated. The hierarchy of the responses was striking similar to those recorded for AhRR. Despite being independent of AhR for their polarisation (Figures S2–S4), the Th1 and Tc1 cells also up-regulated Cyp1A1 in response to exogenous FICZ, but to a lesser extent than Th17/Tc17 cells (Figure 4I, J).

## Discussion

In the investigations described here, dual strategies have been employed to characterise the role played by AhR in the regulation of Tc17 cell development. Both approaches (AhR agonism/antagonism and AhR null mice) have revealed differences in the development of Th17 and Tc17 cells, despite the fact the same cytokine cocktail (IL-1β, IL-6 and TGF-β) is able to drive the induction of both cell types. In line with previous studies [24], [26] it has been shown here that Th17 (but not Th1) cell development requires AhR. Thus, Th17 responses were significantly compromised in AhR^−/−^ mice, and were inhibited by AhR antagonist. Conversely, Th17 responses were enhanced markedly by FICZ in AhR^+/−^ mice. We have shown here that AhR is also required for the development of Tc17 cells. Development of Tc17 cells was reduced in AhR^−/−^ mice, and inhibited by the antagonist in AhR^+/−^ mice. However, in contrast to Th17 cells, Tc17 cells were largely refractory to FICZ, particularly at the level of IL-17 secretion. The implication is that baseline levels of expression of AhR are necessary and sufficient to sustain maximal Tc17 polarisation. As expected, Tc1 development was completely independent of AhR, indeed, during the polarisation of these cells, AhR (and to a lesser extent RORC) was rapidly and substantially down-regulated compared with baseline levels in naïve CD8^+^ precursor cells.

Although the results described here on the role of AhR in Th17 polarisation are largely consistent with those reported previously, there are some intriguing differences. Thus, Veldhoen *et al* [26] demonstrated that availability of natural AhR ligands (namely, FICZ) was sufficient in IMDM media (as a result of UV light exposure from natural sunlight or laboratory lighting [26], [30], [31]) to stimulate IL-22 expression by Th17 cells. In contrast, in our hands, supplementation of IMDM media with additional FICZ was required for optimal IL-22 expression. The inference being that there exists inter-laboratory variation in storage conditions may impact on the AhR ligand content of media. It could be argued that such differences might be attributable to the use in our studies of heterozygous AhR^+/−^ control mice on a C57BL/6J background, rather than wild type C57BL/6 mice as used by Veldhoen *et al* [26]. Consistent with this is the fact that loss of a single AhR allele can have effects on regulation of blood pressure and heme metabolism [32], [33]. Furthermore, reduced IL-17^+^ Th cell numbers have been reported in AhR^+/−^ mice compared with wild type controls [34], although it should be noted that baseline levels were very low in that series of experiments (<10%). In order to address this question directly, comparisons were made of IL-17 polarisation in cells derived from AhR^−/−^, AhR^+/−^ and AhR^+/+^ mice (Figure S5). These experiments revealed that all measures of IL-17 expression (IL-17^+^ cell frequency, IL-17 transcript expression and protein production) were equivalent between AhR^+/+^ and AhR^+/−^ littermates, for both baseline and FICZ inducible responses. In the current series of experiments, therefore, the loss of one AhR allele does not impact on Th17 cell development, and thus AhR^+/−^ littermates are appropriate controls for the AhR null mice.

The primary focus of these investigations was, however, to characterise the role of AhR in Tc17 development. One marked difference between Tc17 cells compared with Th17 cells was the plasticity of the cytokine producing phenotype and the impact of blocking of the AhR on this phenotype. Whereas *in vitro* polarised Th17 cells produced only IL-17, Tc17 cells were found to produce IL-17 and/or IFN-γ. Furthermore, Tc17 (but not Th17) development *in vitro* was associated with initial up-regulation of the type 1 master regulator, T-bet. This is consistent with previous reports of the existence of IL-17/IFN-γ double positive Tc17 cells in mice, albeit with considerably lower frequencies than those reported here [12]. Strikingly, the IL-17 single positive population of Tc17 cells was unresponsive to either stimulation or antagonism of AhR, whereas the IL-17/IFN-γ double positive cells were susceptible to both, converting to exclusive IFN-γ expression when the AhR was blocked. This functional plasticity driven by AhR activation may provide for a fine tuning mechanism *in vivo*, permitting effector activity to be tailored according to local need in response to microenvironmental cues. Plasticity of IL-17 expressing cells (primarily Th17 cells) has been demonstrated previously *in vivo* in mouse models of autoimmune disease, particularly in chronic inflammatory conditions, or during pathogenic challenge [16], [17], [35]. Th17 conversion to IL-17/IFN-γ double positive cells and ultimately IFN-γ producing Th1 cells (designated exTh17) has been shown to be dependent upon IL-23 [16]. Whereas IL-12, rather than IL-23, has been shown to permit the conversion of mouse IL-17-producing Tc17 cells to IL-17/IFN-γ-double producing Tc17/IFN-γ cells [12], [36]. Furthermore, cells that coproduce IL-17A and Tc1 cytokines (IL-2, IFN-γ and TNF-α) in response to *Salmonella Typhi* have been identified in humans, indicating that the distinction between Tc17 and Tc1 responses is not as clearly differentiated as suggested previously and that such cells may be important in protection [37]. We demonstrate here that Tc17 cells can develop into IL-17/IFN-γ expressing cells in the absence of exogenous IL-12, and that the conversion to exclusive IFN-γ producers can be influenced by AhR modulation, providing a further potential level of regulation for such cells *in vivo*.

There were a number of other differences between Th17 and Tc17 cells, including the fact that Tc17 polarisation, unlike Th17 activation, was not associated with increased expression of AhR, and was relatively refractory to exogenous FICZ. The Tc17 cells were sensitive to AhR stimulation as Cyp1A1, a biomarker of AhR activation, was noticeably up-regulated. We speculated that differential regulation of AhR in CD4^+^ and CD8^+^ cells might be driven through variable expression of AhRR, which is involved in a negative feedback loop [28]. However, apart from a slightly more rapid induction of the repressor in Tc17 cells, maximal levels and persistence of stimulation were very similar between the two cell types. Regardless of the potential mechanism, the results demonstrate that Th17 cells are up-regulated more effectively through the AhR and are more responsive to the natural ligand FICZ than are their Tc17 counterparts. This mechanism in CD4^+^ cells may be necessary to ensure the robust differentiation of Th17 cells. Unlike the skewing of Th1 and Th2 cells, where their respective major cytokines (IFN-*γ* and IL-4) act as self-amplifying immunomodulators, it is IL-21, not the major cytokine IL-17, that acts in autocrine fashion on differentiation of Th17 cells. Further amplification through AhR therefore provides a supplementary mechanism through which vigorous Th17 responses are maintained.

Given the increased interest in the role of the AhR in orchestrating Th17 cell responses, it is not surprising that it is being considered currently as a therapeutic target in a range of Th17 autoimmune diseases [38], [39]. However, in mouse models of psoriasis blocking of the AhR unexpectedly resulted in exacerbation of the disease [40]. In the light of the data presented herein, it is possible that this finding might be related to Tc17 responses, as blocking the AhR may result in the acquisition of an exTc17 phenotype, with these cells having lytic ability[17], which could cause disease progression. Interestingly, there are a number of other examples of autoimmune disorders that were initially attributed to Th17 cell pathogenesis that may in fact be elicited, at least partly, by Tc17 cells. These include experimental (mouse) models of autoimmune uveoretinitis, autoimmune encephalomyelitis and hepatic fibrosis and clinical studies in which Tc17 cells have been identified in peripheral blood or lesions, including rheumatoid arthritis and multiple sclerosis [15], [41]–[43].

In conclusion, Tc17 cells have been shown to exhibit identical cytokine requirements for polarisation to their Th17 counterparts. Additionally, AhR plays a role in Tc17 development. However, although baseline AhR expression is required for optimal Tc17 development, these cells do not up-regulate the receptor during development, and do not respond vigorously to high levels of endogenous ligand. Thus, maximal Th17 cell responses are more dependent upon AhR activation than is Tc17 cell development, suggesting that endogenous AhR ligands play a much greater role in driving Th17 cell responses. Furthermore, Tc17 cells display greater plasticity than do Th17 cells, and may be able, therefore, to respond to different microenvironments by switching between Tc1 and Tc17 phenotypes.

## Supporting Information

Figure S1 Gating strategy for *in vitro* polarised Th1/Tc1 cells. Naive CD4^+^ (A) or CD8^+^ (B) (1.25 ×10^5^/ml) cells from AhR heterozygote controls were cultured for 5 days under Th1/Tc1 (IL-12) polarising conditions. After 5 days in culture cells were stimulated with PdBU and ionomycin in the presence of brefeldin A. The polarised cells were then permeabilised and labelled using fluorescent anti-IL-17A (PE) and -IFN-γ (APC) labelled antibodies in combination with appropriate isotype controls. Cells (25000) were analysed by flow cytometry. Results from a representative experiment are displayed as gated analyses. An identical gating strategy was employed for the AhR^-/-^ mice (data not shown).
(TIFF)

Figure S2 The effect of AhR modulation on Th1 and Tc1 cell frequency. Naïve CD4^+^ cells from AhR^+/-^ (A) or AhR^-/-^ mice (B) and naive CD8^+^ cells from AhR^+/-^ (C) or AhR^-/-^ mice (D) were polarised under Th1/Tc1 conditions for 5 days. The cells were cultured in the presence of AhR antagonist (CH-223191) (white bar) or AhR agonist (FICZ) (grey bar) both formulated in DMSO or with an equivalent amount of DMSO alone (black bar). Cells were then stimulated with PdBU and ionomycin in the presence of brefeldin A, followed by permeabilisation and staining with fluorescent-labelled anti-IL-17A (PE [Y-axis]) and -IFN-γ (APC [X-axis]) labelled antibodies. Cells (25000) were analysed by flow cytometry. The percentage of cells positive for IL-17, IL-17 and IFN-γ or IFN-γ alone was calculated by subtracting the isotype controls from the stained cells in each quadrant. Representative quadrant analyses are shown (A-D) and percentage positive cells (E) are displayed as mean ± SE for n = 3 independent experiments. The statistical significance of differences between DMSO control and AhR antagonist/agonist treated cells and of differences between AhR^+/-^ and AhR^-/-^ mice was analysed by one-way ANOVA. No significant differences were recorded.
(TIF)

Figure S3 Cytokine mRNA and protein expression profiles of Th1 cells : effect of AhR modulation. Naïve CD4^+^ cells from AhR+/− (black bar) or AhR−/− mice (white bar) were polarised under Th1 conditions for 5 days. The cells were cultured in the presence of AhR antagonist (CH-223191) or AhR agonist (FICZ) both formulated in DMSO or with an equivalent amount of DMSO alone. Total RNA was isolated and levels of mRNA transcripts for IFN-γ, IL-17A and IL-22 were analysed using RT-PCR and the ΔΔ Ct method (A, C and E). Results were normalised against naive CD4^+^ cells and the housekeeping gene HPRT. Supernatants were also analysed for secreted cytokine by ELISA (B, D and F). Results are shown as mean ± SE for n = 3 independent experiments. The statistical significance of differences between DMSO control and AhR antagonist/agonist was analysed by one-way ANOVA. **, *p*<0.01.
(TIF)

Figure S4 Cytokine mRNA and protein expression profiles of Tc1 cells: effect of AhR modulation. Naïve CD8^+^ cells from AhR+/− (black bar) or AhR−/− mice (white bar) were polarised under Th1/Tc1 conditions for 5 days. The cells were cultured in the presence of AhR antagonist (CH-223191) or AhR agonist (FICZ) both formulated in DMSO or with an equivalent amount of DMSO alone. Total RNA was isolated and levels of mRNA transcripts for IFN-γ, IL-17A and IL-22 were analysed using RT-PCR and the ΔΔ Ct method (A, C and E). Results were normalised against naive CD8^+^ cells and the housekeeping gene HPRT. Supernatants were also analysed for secreted cytokine by ELISA (B, D and F). The statistical significance of differences between DMSO control and AhR antagonist/agonist treated cells was analysed by one-way ANOVA. No significant differences were recorded.
(TIF)

Figure S5 Th17 polarisation and impact of exogenous FICZ : role of AhR phenotype. Naive CD4^+^ cells from wild type, AhR^+/−^ and AhR^−/−^ mice were cultured for 5 days under Th17 (IL-6, TGF-β and IL-1β) polarising conditions in the presence of the AhR agonist FICZ (▪) (300 nM) or DMSO vehicle alone (•). Cells were stimulated with PdBU and ionomycin in the presence of brefeldin A. The polarised cells were then permeabilised and labelled using fluorescent anti-IL-17A (PE) labelled antibodies. Cells (25000) were analysed by flow cytometry and are shown as percentage IL-17A positive for each condition (A). Changes in IL-17A mRNA levels were analysed using RT-PCR and the ΔΔCt method, normalised against naive CD4^+^ cells and the housekeeping gene HPRT (B). Concentrations of IL-17A were analysed by ELISA in supernatants prepared following 5 days culture of the CD4^+^ cells (C). Results are displayed as individual animals (n = 3). The statistical significance of differences between DMSO control and AhR agonist treated cells were analysed by one-way ANOVA. **, *p*<0.01, ***, *p*<0.001.
(TIF)
