# Supplementary figures and images for: The Aryl Hydrocarbon Receptor: Differential Contribution to T Helper 17 and T Cytotoxic 17 Cell Development
Source: PLoS One. 2014 Sep 9;9(9):e106955. doi: 10.1371/journal.pone.0106955 (PMC4159274; doi:10.1371/journal.pone.0106955)

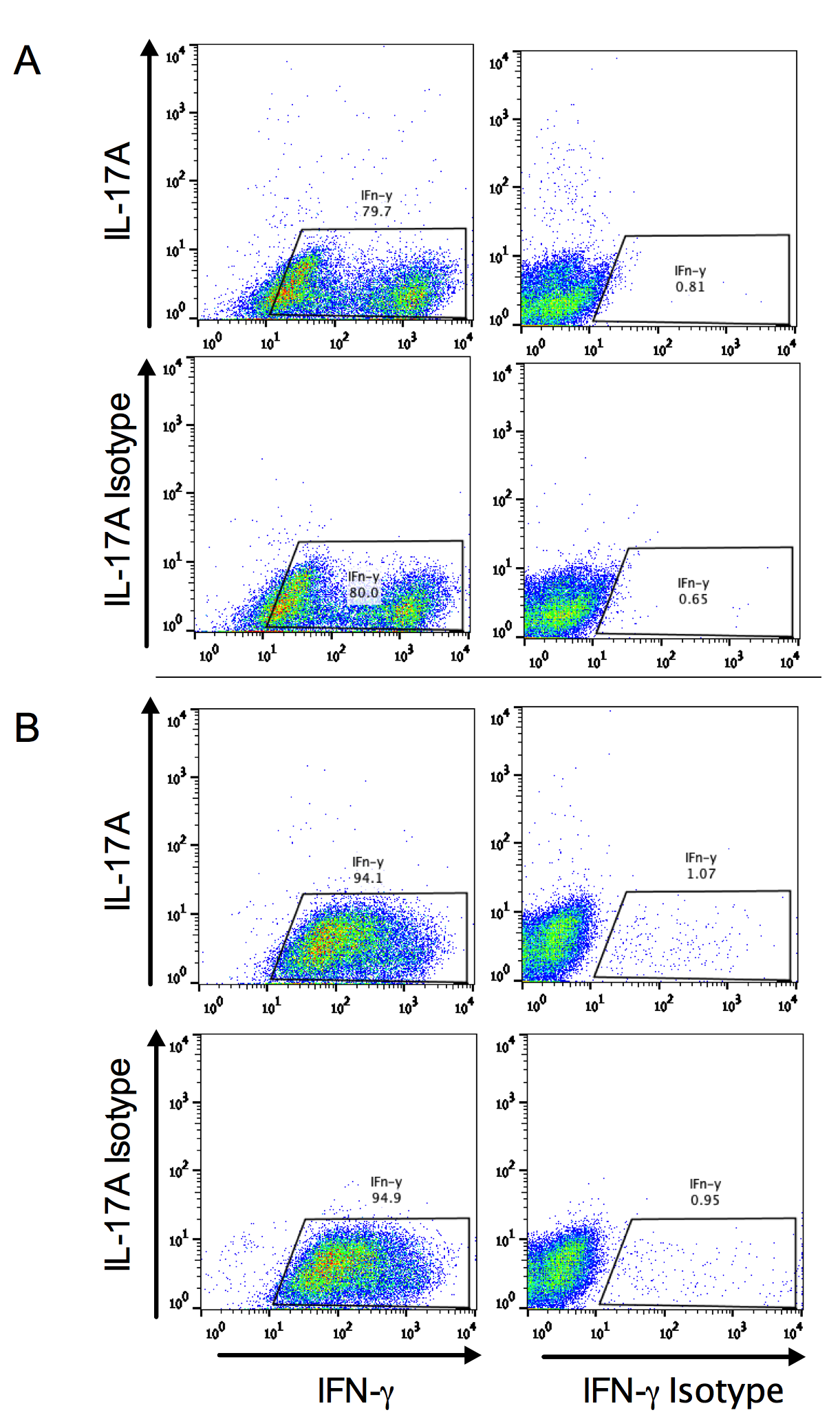

Supplement: Figure S1 — Gating strategy for in vitro polarised Th1/Tc1 cells. Naive CD4+ (A) or CD8+ (B) (1.25 ×10^5^/ml) cells from AhR heterozygote controls were cultured for 5 days under Th1/Tc1 (IL-12) polarising conditions. After 5 days in culture cells were stimulated with PdBU and ionomycin in the presence of brefeldin A. The polarised cells were then permeabilised and labelled using fluorescent anti-IL-17A (PE) and -IFN-γ (APC) labelled antibodies in combination with appropriate isotype controls. Cells (25000) were analysed by flow cytometry. Results from a representative experiment are displayed as gated analyses. An identical gating strategy was employed for the AhR-/- mice (data not shown). (TIFF) [file pone.0106955.s001.tiff]

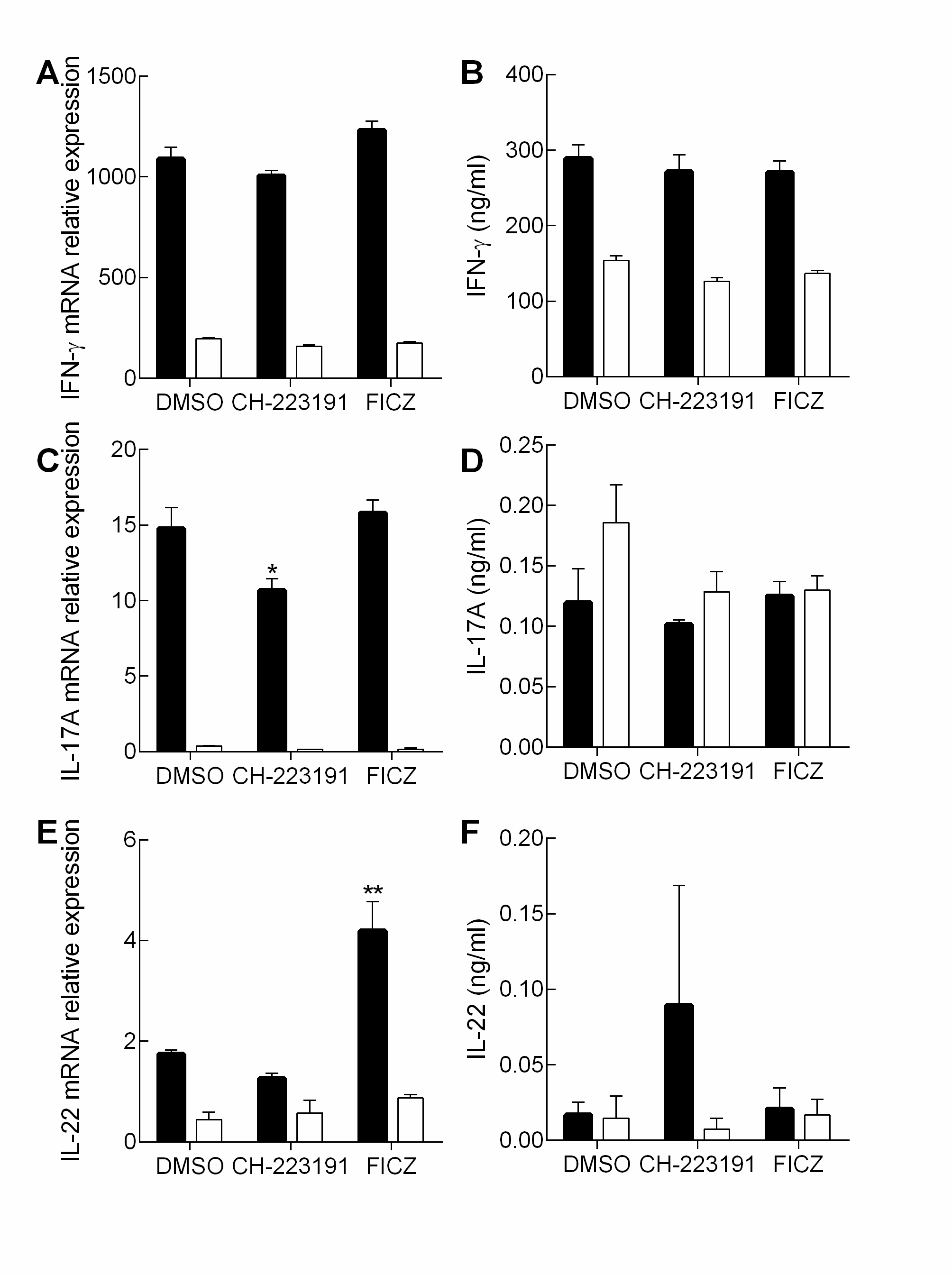

Supplement: Figure S3 — Cytokine mRNA and protein expression profiles of Th1 cells : effect of AhR modulation. Naïve CD4+ cells from AhR+/− (black bar) or AhR−/− mice (white bar) were polarised under Th1 conditions for 5 days. The cells were cultured in the presence of AhR antagonist (CH-223191) or AhR agonist (FICZ) both formulated in DMSO or with an equivalent amount of DMSO alone. Total RNA was isolated and levels of mRNA transcripts for IFN-γ, IL-17A and IL-22 were analysed using RT-PCR and the ΔΔ Ct method (A, C and E). Results were normalised against naive CD4+ cells and the housekeeping gene HPRT. Supernatants were also analysed for secreted cytokine by ELISA (B, D and F). Results are shown as mean ± SE for n = 3 independent experiments. The statistical significance of differences between DMSO control and AhR antagonist/agonist was analysed by one-way ANOVA. **, p<0.01. (TIF) [file pone.0106955.s003.tif]

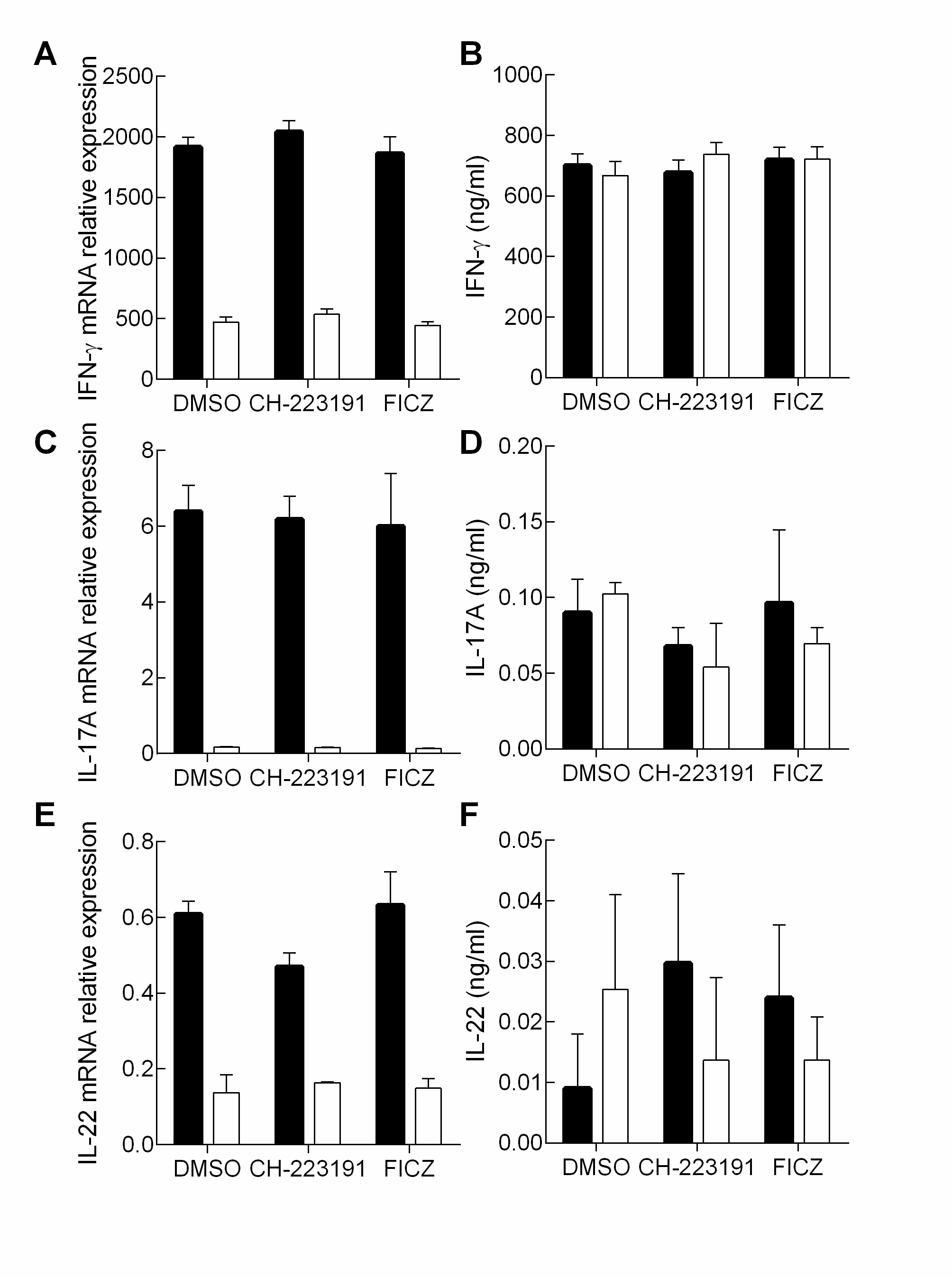

Supplement: Figure S4 — Cytokine mRNA and protein expression profiles of Tc1 cells: effect of AhR modulation. Naïve CD8+ cells from AhR+/− (black bar) or AhR−/− mice (white bar) were polarised under Th1/Tc1 conditions for 5 days. The cells were cultured in the presence of AhR antagonist (CH-223191) or AhR agonist (FICZ) both formulated in DMSO or with an equivalent amount of DMSO alone. Total RNA was isolated and levels of mRNA transcripts for IFN-γ, IL-17A and IL-22 were analysed using RT-PCR and the ΔΔ Ct method (A, C and E). Results were normalised against naive CD8+ cells and the housekeeping gene HPRT. Supernatants were also analysed for secreted cytokine by ELISA (B, D and F). The statistical significance of differences between DMSO control and AhR antagonist/agonist treated cells was analysed by one-way ANOVA. No significant differences were recorded. (TIF) [file pone.0106955.s004.tif]

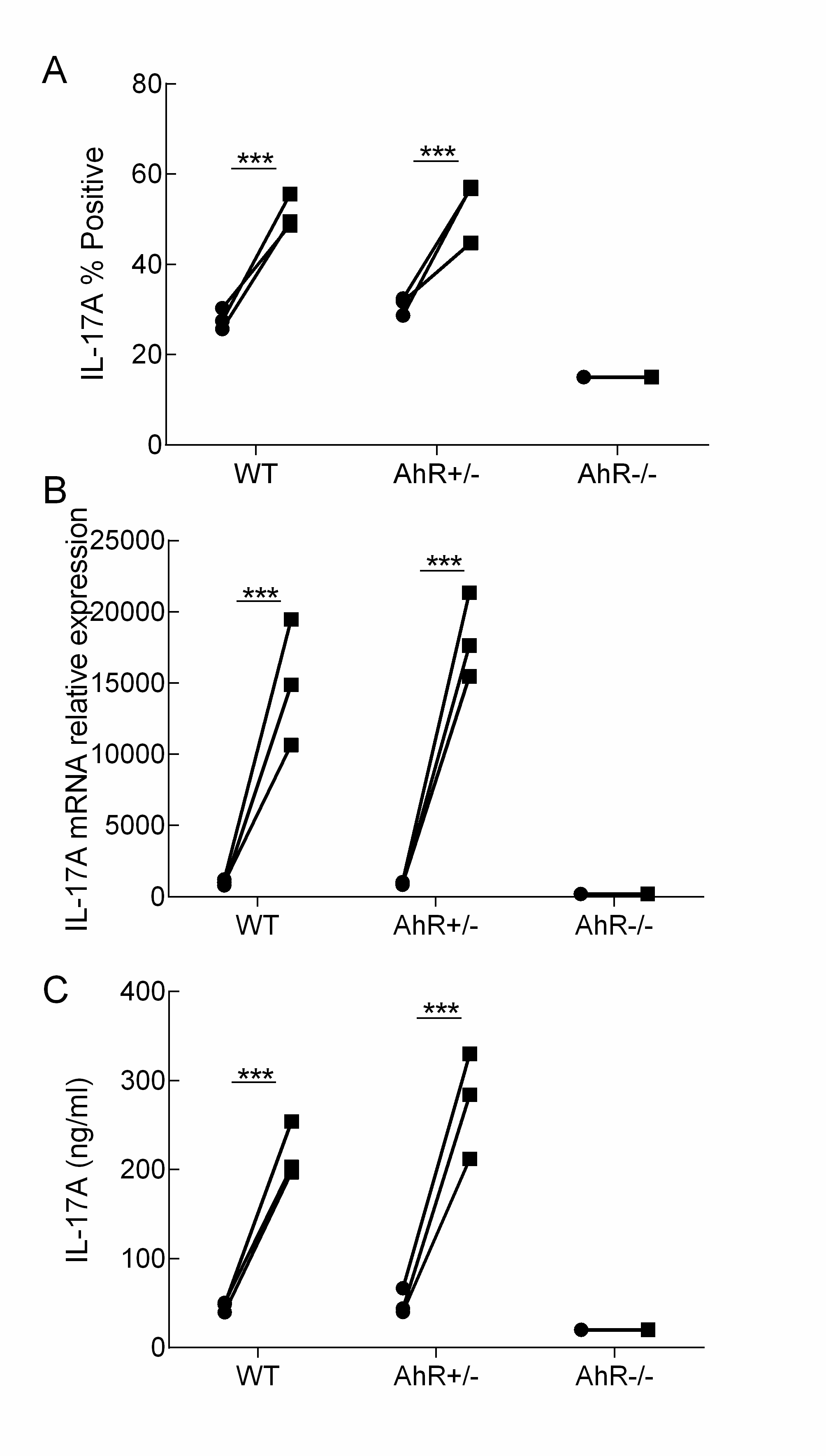

Supplement: Figure S5 — Th17 polarisation and impact of exogenous FICZ : role of AhR phenotype. Naive CD4+ cells from wild type, AhR+/− and AhR−/− mice were cultured for 5 days under Th17 (IL-6, TGF-β and IL-1β) polarising conditions in the presence of the AhR agonist FICZ (▪) (300 nM) or DMSO vehicle alone (•). Cells were stimulated with PdBU and ionomycin in the presence of brefeldin A. The polarised cells were then permeabilised and labelled using fluorescent anti-IL-17A (PE) labelled antibodies. Cells (25000) were analysed by flow cytometry and are shown as percentage IL-17A positive for each condition (A). Changes in IL-17A mRNA levels were analysed using RT-PCR and the ΔΔCt method, normalised against naive CD4+ cells and the housekeeping gene HPRT (B). Concentrations of IL-17A were analysed by ELISA in supernatants prepared following 5 days culture of the CD4+ cells (C). Results are displayed as individual animals (n = 3). The statistical significance of differences between DMSO control and AhR agonist treated cells were analysed by one-way ANOVA. **, p<0.01, ***, p<0.001. (TIF) [file pone.0106955.s005.tif]
